# *QuickStats:* Percentage* of Adults Aged ≥18 Years with Diagnosed Diabetes,^†^ by Urbanization Level^§^ and Age Group — National Health Interview Survey, United States, 2019^¶^

**DOI:** 10.15585/mmwr.mm7018a4

**Published:** 2021-05-07

**Figure:**
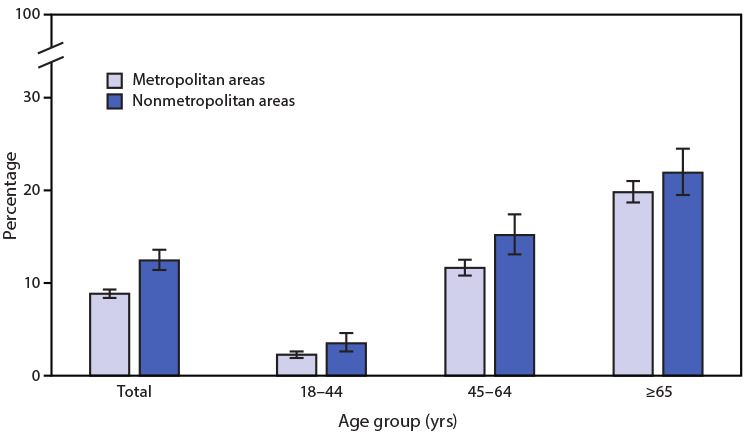
In 2019, the percentage of adults aged ≥18 years with diagnosed diabetes was higher among those living in nonmetropolitan areas (12.4%) than among those living in metropolitan areas (8.9%). Percentages of adults with diagnosed diabetes were higher in nonmetropolitan than metropolitan areas for those aged 18–44 years (3.5% versus 2.3%) and 45–64 years (15.2% versus 11.6%). Among adults aged ≥65 years, the difference by urbanization level (21.9% in nonmetropolitan areas versus 19.8% in metropolitan areas) did not reach statistical significance. The prevalence of diagnosed diabetes increased with age in both nonmetropolitan and metropolitan areas.

**Source:** National Center for Health Statistics, National Health Interview Survey, 2019. https://www.cdc.gov/nchs/nhis.htm

